# Online training in evidence-based medicine and research methods for GP registrars: a mixed-methods evaluation of engagement and impact

**DOI:** 10.1186/s12909-021-02916-0

**Published:** 2021-09-14

**Authors:** Hania Rahimi-Ardabili, Catherine Spooner, Mark F. Harris, Parker Magin, Chun Wah Michael Tam, Siaw-Teng Liaw, Nicholas Zwar

**Affiliations:** 1 Centre for Primary Health Care and Equity, UNSW Sydney, Sydney, NSW 2052 Australia; 2 Research and Evaluation Unit, GP Synergy, Sydney, NSW 2304 Australia; 3 School of Medicine and Public Health, University of Newcastle, Callaghan, NSW 2304 Australia; 4 Primary and Integrated Care Unit, South Western Sydney Local Health District, Liverpool, NSW 2170 Australia; 5 WHO Collaborating Centre (eHealth), School of Population Health, UNSW Sydney, Sydney, NSW 2052 Australia; 6 Faculty of Health Sciences and Medicine, Bond University, Gold Coast, 4229 Australia

**Keywords:** Evidence-based medicine, Online education, GP, Clinical practice, Mixed methods

## Abstract

**Background:**

Evidence-based medicine (EBM) is a core skillset for enhancing the quality and safety of patients’ care. Online EBM education could improve clinicians’ skills in EBM, particularly when it is conducted during vocational training. There are limited studies on the impact of online EBM training on clinical practice among general practitioner (GP) registrars (trainees in specialist general practice). We aimed to describe and evaluate the acceptability, utility, satisfaction and applicability of the GP registrars experience with the online course. The course was developed by content-matter experts with educational designers to encompass effective teaching methods (e.g. it was interactive and used multiple teaching methods).

**Methods:**

Mixed-method data collection was conducted after individual registrars’ completion of the course. The course comprised six modules that aimed to increase knowledge of research methods and application of EBM skills to everyday practice. GP registrars who completed the online course during 2016–2020 were invited to complete an online survey about their experience and satisfaction with the course. Those who completed the course within the six months prior to data collection were invited to participate in semi-structured phone interviews about their experience with the course and the impact of the course on clinical practice. A thematic analysis approach was used to analyse the data from qualitative interviews.

**Results:**

The data showed the registrars were generally positive towards the course and the concept of EBM. They stated that the course improved their confidence, knowledge, and skills and consequently impacted their practice. The students perceived the course increased their understanding of EBM with a Cohen’s d of 1.6. Registrars identified factors that influenced the impact of the course. Of those, some were GP-related including their perception of EBM, and being comfortable with what they already learnt; some were work-place related such as time, the influence of supervisors, access to resources; and one was related to patient preferences.

**Conclusions:**

This study showed that GP registrars who attended the online course reported that it improved their knowledge, confidence, skill and practice of EBM over the period of three months. The study highlights the supervisor’s role on GP registrars’ ability in translating the EBM skills learnt in to practice and suggests exploring the effect of EBM training for supervisors.

**Supplementary Information:**

The online version contains supplementary material available at 10.1186/s12909-021-02916-0.

## Introduction

Evidence-based medicine (EBM) is the integration of best available clinical evidence with clinical expertise and patient values to inform a clinical decision [1]. It is recognised as a core skillset for improving the quality and safety of health care [2]. This is acknowledged by the inclusion of EBM competencies in the curriculum for general practice education throughout the training continuum - from undergraduate learners to continuing professional development programs for established general practitioners (GPs) [3, 4].

Despite some evidence that GPs generally acknowledge the importance of EBM [5], it is not always practiced in routine care [6]. For instance, Australian GPs and GP registrars (trainees in specialist general practice) have been observed to prescribe antibiotics for respiratory tract infections in a non-evidence-based manner [7, 8].

There are a range of influences on the use of EBM in practice. Many of these factors are shared among GPs and GP registrars. Some of these barriers are at the GP-level, such as doubt about the applicability of the research evidence to practice [9, 10]) or lack of skills in finding, appraising and applying evidence [5, 6, 11–13]. Some are workplace-related such as the influence of previous practice or peers [9] and resource constraints (e.g. time pressure, access to resources such as reliable internet or subscriptions to sources) [5, 6, 11–14]. Patient factors also influence clinical practice. While patient-centred care is an important feature of high-quality care [15], navigating between EBM and patient preferences and beliefs for treatments that are unsupported by evidence, can be challenging [5, 6, 11–13]. In addition, there are some specific barriers related to GP registrars, for example, GP registrars often seek answers for their clinical questions, especially for more complex ones, [16] by consulting supervisors and colleagues [17]. This could be a barrier or facilitator to the practice of EBM, depending upon the supervisor [18, 19], Some studies also reported that the atmosphere established by supervisors (authoritarian vs collaborative) could be a barrier for GP registrars [20].

Medical education has a major role in preparing a workforce skilled in EBM, but its translation from learning to practice is challenging [21, 22]. Specific EBM teaching strategies such as clinically integrated training, problem-based learning, and e-learning can improve knowledge, attitudes, and skills in undergraduate medical students [21]. However, there is limited evidence that these strategies directly influence clinical practice [21].

Internet-based learning activities offer a range of advantages, such as ease of access from various settings and for a large audience [23]. Online training can be efficient compared to the traditional face-to-face methods as it allows distance learning when local training opportunities and resources are limited; provides convenience and flexible learning; reduces travel time and expenses for learners [24]. It can be beneficial and time-efficient when trainees have diverse ranges of background knowledge and customised course content is needed [25]. These features make online training a pragmatic method for training medical professionals in full-time clinical practice. A recent review of 14 studies concluded that online training can be as effective as other alternative methods for increasing knowledge and improving clinical practice of medical professionals [23].

While online learning has potential benefits, it must engage and empower learners for the learnings to be applied in the real world. Boettcher’s review of pedagogical theory and research identified the following core principles for effective teaching (traditional and technology-enhanced): that teaching should be interactive, customised to learners’ background knowledge, deliver information in the form of organised chunks, and provide opportunities for learners to write, explain and analyse [26]. EBM educational interventions that use a variety of learning methods (e.g. video, written materials) are more likely to have a significant effect on learning EBM than those that used a single method [27, 28]. More broadly, training should be relevant to practice [29], case-based [30, 31] and implemented when learners are exposed to clinical cases to practice the skills learnt [29].

While there is abundant evidence for the efficacy of online learning, the teaching of EBM is a singular area. There is a good deal of theoretical understanding to be imparted, but also a complex application of that theory to practical use (our course was designed to equip and encourage our registrars to apply EBM techniques to their clinical practice). There is a modest amount of evidence for online EBM courses [21, 32–35]. We are not aware of studies of online EBM training for GPs. Teaching EBM to GPs and GP vocational trainees is teaching in a unique and problematic environment. GPs must function in an environment of much greater diagnostic uncertainty than medical specialty practice. This creates particular difficulties in the application of evidence to practice. Also, much of the evidence on which GPs must rely in practising EBM has been derived in non-GP populations (specialist or hospital practice). Interpreting evidence and applying it to practice provides another difficult layer to teaching EBM to GPs and GP registrars. Finally, there is still a sizable minority of GP registrars who have considerable indifference to, if not resistance to, EBM. A preference for personal experience or the opinion of superiors rather than research-derived evidence is quite common and may be fostered in some environments within the apprenticeship model training system of Australian GPs. Our course addressed these issues of the importance and relevance of practising EBM in general practice, how EBM can be practised in an environment of heightened diagnostic uncertainty, and how evidence should be critically appraised for its relevance to a particular general practice patient.

### The study objectives

This research explored GP registrars’ experience with an online interactive training course that aimed to increase knowledge of research methods by GP registrars and, ultimately, may be able to produce practitioners who can use evidence in practice. The findings of this study not only can be used for the improvement of the current training course but also for informing future similar course development by identifying its efficacy, strengths and limitations. The study’s research questions were:

1. What were the GP registrars’ experiences with the course (including how engaged were they with the course)?
2. What were the impacts of the course on GP registrars’ attitudes, knowledge, skills and clinical practice?
3. What factors influenced the impact of the course on the GP registrars’ clinical practice?

## Methods

### Design

The evaluation was a one-group design (post-test only) conducted after completion of the course, using a triangulation mixed methods design.

### Recruitment and sample

Participants were GP registrars who completed the online course. GP registrars are required to complete the course by the end of term two of the training. GP registrars who completed the course during Nov 2016-Feb 2020 (*n* = 1142) were invited to fill in an online survey via a link within the course.

All GP registrars who completed the course within the six months prior to data collection (2019–2020) were invited to participate in semi-structured interviews. Registrars were invited to express interest in being interviewed for the study via:

- An invitation within the online survey used for course satisfaction data collection.
- An invitation from GP Synergy, sent to registrars on completion of the course and to registrars who completed the course since 1st January 2019
- An invitation within the online course.

Sampling was purposive, based on potential participants’ characteristics of medical training (Australia vs overseas) and GP training pathway (general vs rural).

### Intervention

An online education course (“the course”) was designed and built for GP registrars to complete as a compulsory component of their vocational specialist training. This was commissioned by GP Synergy Ltd., the government-funded vocational GP regional training organisation in New South Wales and the Australian Capital Territory, Australia. The reason for developing the course was that GP Synergy needed a scalable method of teaching GP registrars (annual intake of 500 registrars) spread over the whole of New South Wales and the Australian Capital Territory (an area over 800,000 km^2^).

The course was developed by content and education experts from UNSW Australia (including academic GPs) and an interactive software developer (Smart Sparrow Pty Ltd) to ensure modules were educationally sound.

The course was designed to provide a grounding in research (quantitative and qualitative), epidemiology and critical evaluation and how evidence from these sources is incorporated into clinical practice: that is, the course sought to present the knowledge required to practice as an evidence- based practitioner. The overarching aim of the course was “to have registrars using evidence and practising EBM in their everyday practice” [36]. It comprised six modules of critical thinking and ethical principles in human research, critical appraisal skills, exploring the evidence on a clinical question including systematic review and meta-analysis, clinical epidemiology, quantitative research methods and qualitative research methods. Each module was divided into three-to-six lessons with lesson-specific learning objectives (see Additional file 1, Table S1). The course aimed to enable participants to:

1. identify links between research and practice
2. apply the research findings to examples from practice
3. describe the principles of research design
4. participate in research.

The modules included text with visual learning methods such as graphs and images, video lectures, links to publicly accessible video resources, interactive exercises (e.g. multiple choice), clinical scenarios and further reading. To relate the course content to GP registrars’ daily practice, clinical scenarios were included in the lessons. Each module was expected to take about an hour to complete, not counting additional reading. Fig. S1 illustrates some examples of the course screens on how interactivity and engaging elements delivered.

### Instruments and data collection

The online survey asked participants to indicate the degree to which they agreed with 11 statements that described their experience with the course using a 6-point rating scale; rate their understanding of the topic before the course and after completing the course on a scale from 0 to 10; and answer two open-ended questions: 1) what they liked most about the course and 2) what they would like to see changed. Participants self-completed the online survey via the link provided in the course.

The semi-structured interview schedule (Table 1) contained two sections:

1. Experience with the course
2. Impact of the course on clinical practice.

**Table 1:** Interview questions

Number	Question
1	What are your thoughts on the program?
2	If you didn’t complete the program, can you provide feedback on why?
3	How engaging were the online modules?
4	How easy was it to use the online modules?
5	Was the content pitched at the right level of difficulty and detail?
6	Were your learning needs and expectations met?
7	How relevant were the topics to you as a general practitioner?
8	We are keen to determine whether you are using the EBM skills learnt in the modules in your practice.
9	Has completing the program led to you considering further training in research?

Interviews were conducted by telephone in one or two sessions, depending on the course completion time:

1. Two sessions: If the Registrar had completed the course less than one month before recruitment, the first interview was restricted to section one of the interview schedule. A second interview to complete section two of the interview schedule was conducted three months later.
2. One session: If the Registrar had completed the course at least three months before recruitment both sections of the interview schedule were completed in the one interview.

The timing was designed to allow participants the opportunity to apply the knowledge and skills gained during the course before answering the questions in section two about how the course influenced clinical practice.

The interviews were conducted via telephone by a trained Research Officer [HR-A] who had not been involved in the course development. The average times to complete the interviews were 23 min (first interviews) and 19 min (second interviews).

### Data analysis

Descriptive statistics were used to present quantitative data from the online survey. The difference between the self-report level of understating of the topic before and after the course was tested using paired t-test and the effect size compared using Cohen’s *d*. Data analysis was performed using SPSS (version 23). The answers to open-ended questions were coded and summarised. These were then discussed between authors [CS, HR-A] and grouped into broader categories and sub-categories based on their similarities and differences.

Data collection and analysis were concurrent and iterative for interviews. The interviews were transcribed verbatim and coded using the software program NVivo (Version 12 Pro). A thematic analysis approach was used [37] to analyse the data from qualitative interviews. The initial few transcripts of both the first and second interviews were open coded by pairs of researchers and the codes compared and discussed among five authors [CS, MH, HR-A, CT, PM]. Later codes were updated based on the discussion, and the rest of the transcripts were coded by one of the authors [HR-A]. In all steps of coding, the analyst constantly refined the earlier codes in the light of newly emerged codes. When all data were coded, the generated themes and codes were again discussed among all authors, until consensus was reached. We aimed to achieve thematic saturation but due to COVID-19 (as GPs, their capacity to find free time for the interview was affected by the high demand for health professionals as well as the stress and upheaval relating to the education program moving suddenly online and their practices’ structural responses to the pandemic), data collection was ceased before the data saturation was complete. There was still good saturation in most of the themes.

To ensure trustworthiness, an audit trail of all the steps and decisions made during the research process were recorded [38]. The researcher triangulation method [38] was used by involving multiple analysts to bring different perspectives into the findings. Verbatim quotations of various participants were provided to support the study findings. Considering reflexivity, most of the authors (NZ, MH, CT, PM, TL) were academic GPs in current clinical practice, and all authors except one (HR-A) were experienced and involved in activities relevant to teaching EBM to GPs. All authors had prior experience in analysing qualitative data. The main analyst [HR-A] kept a reflexivity journal during analysis to discuss it with all authors.

When data analyses were completed, qualitative and quantitative findings were synthesised to answer the study questions. Codes were reviewed for patterns, and they were further revised and developed into themes to answer the research questions. The final themes are presented in Fig. 3 in the result section.

## Results

### Participants

**Fig. 1:**
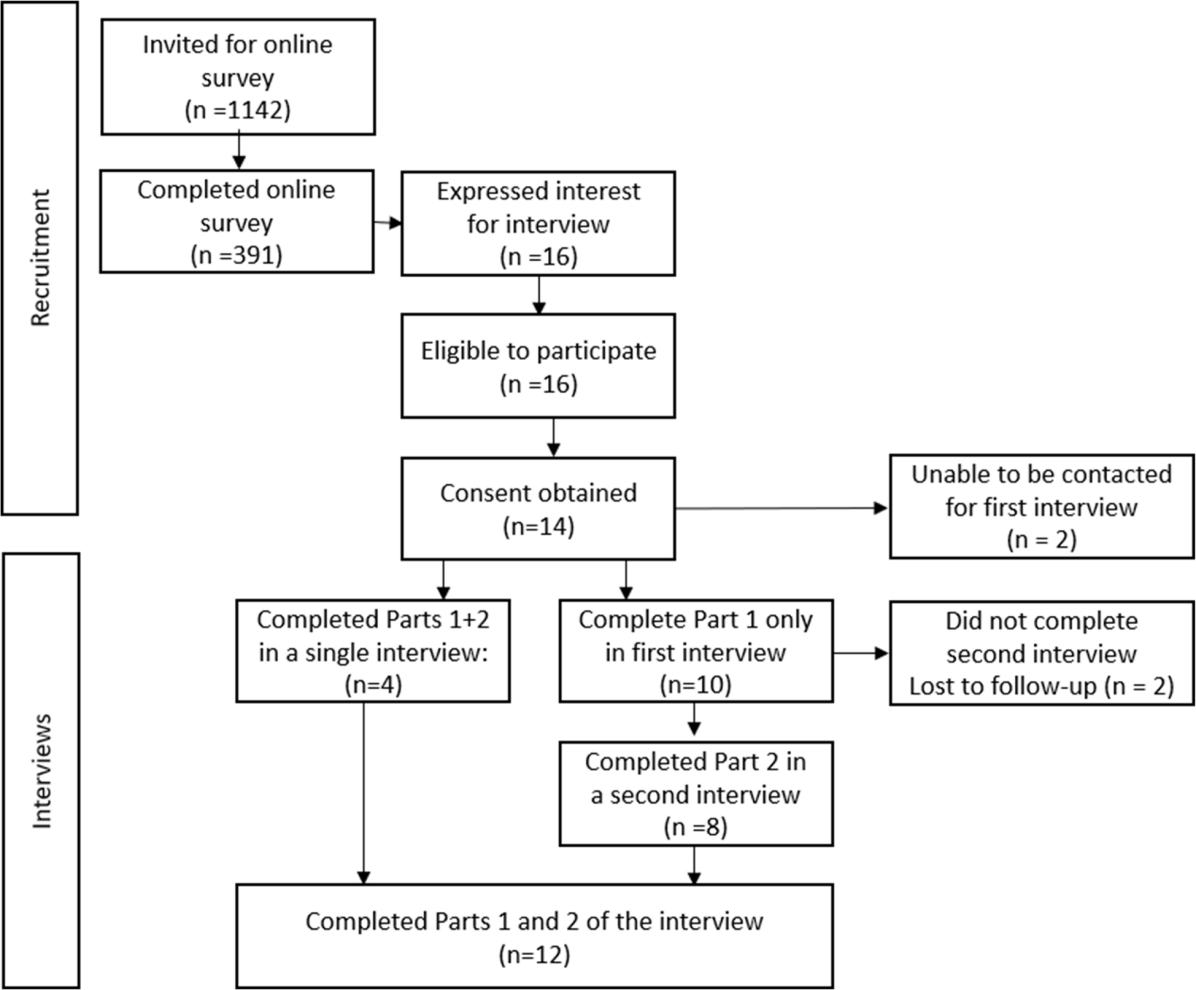
Recruitment and interview process

Of 1142 GP registrars who invited to take part in the online survey, 391 (34%) completed the survey between Nov 2016-Feb 2020. Twelve GP registrars completed interviews. Details of the recruitment process and data collection are provided in Fig. 1.

The interview sample comprised nine women, five men; two registrars enrolled in the rural pathway, and only one registrar who had completed medical training overseas. The interviewees enrolled in the course during the second or last year of their three-year (full-time equivalent) program. Participants stated that they spent an average of nine hours to complete the whole course.

Results are presented to answer each research question.

### RQ1. Participant experience with the course

**Fig. 2:**
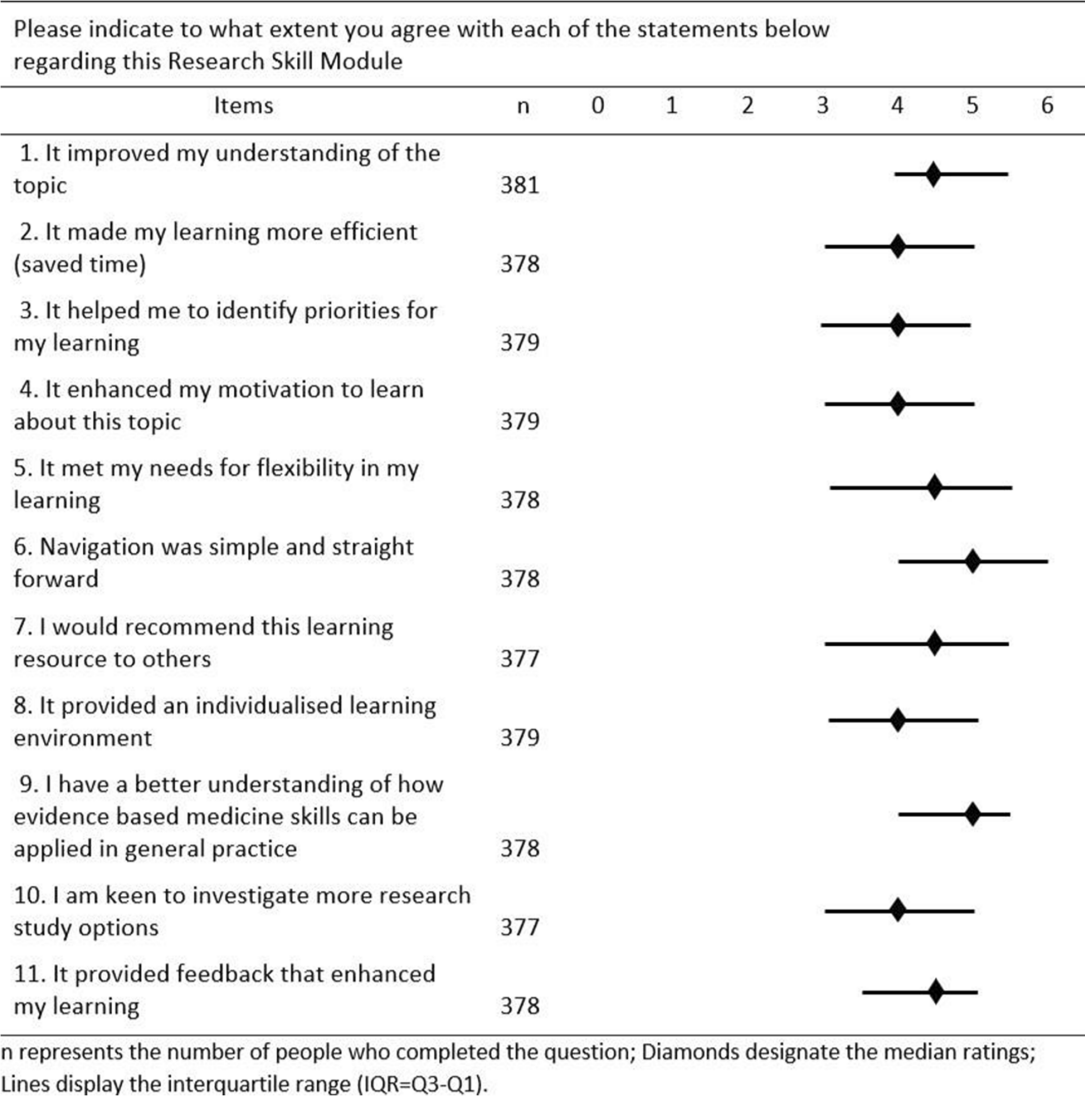
Participants ratings of the course using a 6-point rating scale (*n* = 391)

Registrars’ responses to 11 questions on satisfaction with the course are presented in Fig. 2.

Data from the online survey and the interviews indicated that participants’ course experience was generally positive. As illustrated in Table 2, the triangulated methods provided some unique and some common feedback.

**Table 2:** Participant experience feedback from online survey (*n* = 391) and interviews (*n* = 14)

	Survey multiple choice	Survey open-ended questions	Qualitative interviews
**Content**			
Easy to understand		✓	✓ *I felt like the content was delivered in a way that made it very easy to understand* [#14].
Relevant to practice, particularly/ case studies		✓	✓ *I think it was quite relevant. Especially when they brought in a case study that is very common in the general practice room* [#12].
A good resource		✓	✓ *I think the main things were resources I could use to look up things in future if I had trouble. So, I saved them as bookmarks* [#12].
**Delivery**			
Flexible	✓ (Q.5) Flexibility in learning	✓ Flexible with time and pace	✓ *The fact that it was online, that I could do it in my own time, and that flexibility was great* [#09].
Easy navigation / user friendly	✓ (Q.6)	Mixed	Mixed *The navigating the system was fine. It was easy* [#04]. *I did find a problem with it. When you had to move answers into boxes, if it accidentally went into the wrong box* [#08].
Individualised learning	✓ (Q.8)		
Feedback /interactivity	✓ (Q.11)	✓ Liked the interactivity e.g. quizzes & feedback	✓ *It was good that we were sort of asked to generate a response, to answer a question. I also enjoyed that they [the course] gave you a model answer* [#14].
Engaging		✓	Mixed *So, I thought it was good mix of media which made the presentation interesting and it kept my attention for longer than it may have otherwise [#07].* *I found that it was a little bit difficult to engage with some of the modules [#3].*

The responses to online questions on registrar satisfaction were mostly in the upper half of the rating scale, suggesting general satisfaction (Fig. 2). Consistent with the qualitative data, the participants highly rated the ease of navigation (Q.6), the flexibility of the online delivery (Q.5) and the feedback (Q.11).

In contrast to the positive responses to Q.6, the open-ended question about what participants would like changed (Table S2) identified that many participants had problems with technical issues, and some thought the interface could have been more user friendly.

Data from the open-ended questions (Additional File 1, Tables S2 and S3) and interviews (Table 2) provided additional feedback on the course content: that it was easy to understand, provided relevant examples, it was relevant to practice, and it provided useful resources for future use.

### RQ2. Impacts of the course on attitudes, knowledge skills and clinical practice

Data from the survey and the interviews indicated that the participants thought the course positively increased their confidence, knowledge and skills of EBM. Interview data (presented below) suggested the course changed the participant’s clinical practice to better incorporate EBM.

#### Attitudes

Most participants said they were only interested in the application of research into practice. Only a few participants said that they their interest in doing or participating in research. Was influenced by taking the course.

Participants mostly found themselves more ***confident*** in understanding, interpreting and appraising research evidence after completing the course.

I feel more confident, and I think that I would be able to if I needed to look through a study and make some comments [#08-female].

However, they acknowledged that they might not be able to conduct research, or there is room for improvements.

I probably feel about the same as I did before. I don’t know that I feel any more or less confident [#10-female].

#### Knowledge

An increase in knowledge was identified in the interview and the online survey. Based on the survey findings, participants’ self-reported understanding of the topic substantially improved from the mean of 4.4 (out of 10) ± 0.1 (SD) before the course to 7.2 ± 0.1 after completing the course (*n* = 320, p<0.0001) which represents a very large effect size (*d* = 1.6). Similar findings found from the interview.

It’s a good idea I think to have an understanding of research when we’re going out into clinical practice [#05-female].

It [the course] makes me more aware to incorporate research into my practice [#07-male].

In addition, participants acknowledged that the course, led them to start questioning practice where clinicians follow others’ experience/opinion without thinking critically.

The way it’s [the course] influenced my management is just always being aware that just because it’s been done as usual practice doesn’t necessarily mean like it’s evidence-based and doesn’t mean that it’s necessarily proven to be effective [#01-male].

One participant highlighted an improvement in her understanding of the importance of contextual and environmental factors, including patient preferences in applying evidence.

It’s not just whether or not they’re sick, it’s also about how they approach health providers, … So, I just keep it in the back of my mind that we need to be aware of the other socioeconomic and environmental factors which a lot of that qualitative research helps us consider as well [#14-female].

#### Skills

Some participants said they were now able to critique research evidence and interpret research studies.

I think it’s just helpful to …being able to interpret and synthesise how that can apply [research] to your clinical practice [#03-female].

Some participants reported that they learnt how to frame their clinical questions and find an evidence-based answer for it.

Instead of thinking that you don’t know something, and letting that overwhelm you, you become a little bit better at devising a clinical question and knowing where to answer is [#13_2-male].

#### Clinical practice

Some participants reported that the skills that they learnt had led to some changes in their practices. They used the skills learnt and research evidence to ***investigate an answer to their clinical questions*** particularly when they were dealing with uncertainty such as dealing with a complex case or when a patient queries about a treatment that they have not heard of. They acknowledged that they used skills such as clinical appraisal, particularly the levels of evidence [#3,4,7,14] to interpret study findings, findings that drug representatives presented to them, and guidelines.

Looking at evidence and working out how reliable this is, and then using that to, you know, guide treatment or, you know, not needing to always rely on guidelines to make a better judgment, depending on the clinical situation after reviewing what evidence is available [#08-female].

A few participants indicated that the skills that they learnt in the course ***reaffirmed the importance of communication skills with patients***. Their improved understanding of research findings and being able to elaborate the difference between high quality and low-quality evidence to patients reportedly improved their communication with patients, particularly in instances when a patient had a query regarding different treatments and medications.

I do bring it back to research and how just because one thing works for someone doesn’t mean that it works with you and just break it down that way, and also talking like, high-quality studies versus poor quality as evidence [03-female].

### RQ3: Mediators of impacts (reported barriers and facilitators)

Responses from the qualitative interviews indicated that barriers and facilitators to practicing EBM related to the GP (GPs perceptions of EBM, comfort and priority); the work-place (time, the influence of supervisors, the impact of system and access to resources); and patients (treatment expectation being different from evidence).

#### GP factors

Most of the participants had ***positive attitudes towards EBM*** and acknowledged the value of EBM.

Obviously, we have to practice evidence-based medicine, so in order to do so, we need to be able to understand and interpret and incorporate research into our practice [#07-male].

Almost all participants reported they needed to seek information to inform their decisions on a daily basis. They reported that they tried to choose EBM resources that were recent and relevant to the Australian context and had confidence in the quality of the information.

All of them [the guidelines that I use] are sort of peer-reviewed and accepted by the wider community as factually correct [#11_2-male].

A few participants expressed a different opinion and described research evidence as neither relevant nor transferable into clinical practice.

I think they’re [research evidence] just answering questions that are quite different from the questions that we get in general practice [#06-female].

However, a few participants described an approach based upon trust in the credentials of the source of information:

I do feel if it’s [a research findings] published in a reputable source, I tend to leave it without thinking too critically… I trust my supervisor and feel that they are quite competent [#05-female].

A barrier that was described was, despite that the participants were willing to change, they felt more ***comfortable with what they have already learnt*** and get accustomed to, than using new evidence-based resources.

Often you’re introduced to something like an UpToDate [a resource for supporting clinical decision] quite early, so you get good at searching and using it, that you know what sort of services are on it [#13_2-male].

#### Work-place factors

***Time*** was one of the main barriers identified for accessing and using research evidence in practice by almost all participants. The time-consuming nature of using research evidence was attributed to the way it was accessed, (for example, an initial need for login to the webpage of some organisations such as RACGP), the overwhelming amount of information identified by searches, and the time needed to critically appraise the findings.

If I were to go through Cochrane and look up a whole bunch of different articles which would take a lot longer [#05-female].

For almost all registrars, pre-appraised resources that are brief and ready to use, such as guidelines, were preferred over primary research evidence.

I would tend to use resources that are incorporated study findings into a summary like eTG [online Therapeutic Guidelines], I don’t read the specific articles and therefore analyse the data’ [#5-female].

Participants’ responses indicated that using research evidence might not be their ***priority***; work and exams were specified as activities that they prioritised.

Because I do have exams coming up I haven’t been able to do – look into research papers [#11_2-male].

Participants reported that ***supervisors*** could be ***role models*** influencing GP registrars’ beliefs about evidence-based medicine by encouraging and guiding GP registrars to practice evidence-based medicine. Participants who said their supervisors encouraged them to use research evidence had a stronger belief than other participants about the applicability of research into practice.

Well, I think evidence-based research should inform good clinical practice, and it should always be the starting point for good management… Well, my supervisors have mostly been very evidence-based as well, so I’ve actually just learnt a lot from how they appraise studies [#03-female].

Some participants also indicated that some supervisors expect GP registrars to follow their advice and treatment approach without critically evaluating the relevance of the advice into the clinical situation.

[one of my supervisors is a] real old-school doctor, right, so they are less likely to change or read the literature or – this is the way we’ve always done it, so that’s the way they’ll always do it. So that’s the way they want me to do it as well. Which may not be – it’s not dangerous, but it may not be an optimal solution [#11_2-male].

#### Patient factors

***Patient expectation*** for particular treatment options that were not supported by evidence was reported to be a barrier to practicing EBM.

That’s mainly patient preference, so potentially they don’t want to go with the evidence-based therapy [#04-male].

### Summary

**Fig. 3:**
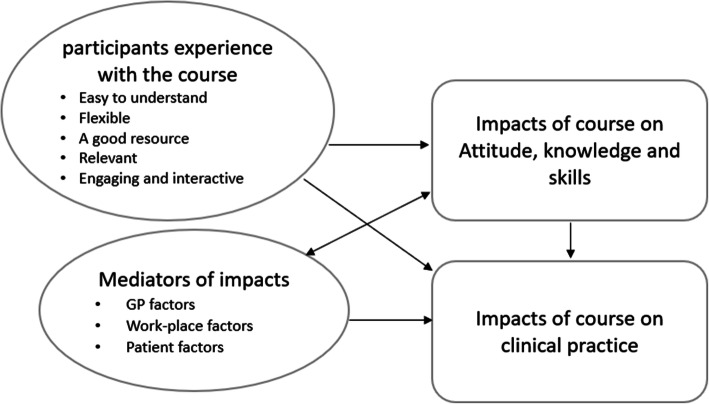
Participants’ experience with the course, perceived course impacts and factors that influenced the outcomes

Figure 3 illustrates the main themes identified to answer the study questions and their interactions. The interactions were interpreted from the qualitative data and whether the specified changes and influences were related to the course. In summary, participants had a positive experience from the course and stated that taking the course led them to improve their, confidence, knowledge, and skills.

Participants specified that they used the skills learnt in the course for interpreting evidence, investigating their clinical questions, and the course led them to have better communication with patients in their clinical practice. The factors that influenced the course’s impact were specified to be related to GP, work-place, and patient factors.

## Discussion

This study evaluated GP registrars’ experience with an online training course that aimed to increase their knowledge of research methods and the application of critical appraisal. The course was a compulsory component of their vocational specialist training developed by content and educational-design experts. The data showed the registrars were generally positive towards the course and the concept of EBM. They stated that the course improved their confidence, knowledge, and skills and consequently impacted how they practice. Particularly, participants specified that they used the skills learnt in the course for investigating their clinical questions, interpreting evidence, and communicating with patients in clinical practice. The self-reported improved understanding of the topic increased with a very large effect size (*d* = 1.6).

Participants in the online survey and qualitative interview reported that they expected a course to be interactive, relevant to practice and multi-media. For example, the course videos were appreciated by participants and acknowledged that it made the course engaging. This is aligned with research findings on EBM education [27–31] and principles of effective teaching [26] that support interactive and case-based learning. For example, Ilic et al. 2015 reported that participants preferred the YouTube videos and found it engaging compared to the lectures delivered for EBM teaching [39]. Similarly, Cranney 2001 reported that GPs who participated in an educational program for evidence-based hypertension management preferred topics that were relevant to their daily practice [11].

Registrars identified multiple barriers and facilitators for implementing EBM. Of those, some were GP-related factors including GPs’ perception about EBM and comfort, some were work-place and system related, including the influence of supervisors, time and access to resources, and one was related to patient preferences. These factors identified in this study were similar to previous research influencing EBM practice [5, 6, 11, 12]. In this study only one participant found PBS as a system barrier for prescribing new evidence-based medicines; however, this has not been identified in earlier studies. Below we further focused and discussed the factors that might be addressed by educational interventions.

Given that GPs’ practice behaviours are established early [40] and remain stable over time, ensuring a supportive work environment for the practicing of EBM is likely to be important for the translation of learning from the course to practice. The influence of supervisors can be utilised to facilitate promoting EBM in practice in future educational interventions. In the current study, participants who were positive about the EBM stated that their supervisors had advised using research evidence in their practice. One of the ways that GPs registrars learn about prescribing is through teaching sessions and discussions with supervisors [41]. Previous studies have also shown that GP registrars’ practices are likely to be influenced by the decisions of senior supervisors [18], and their practicing behaviours are likely to be similar to their supervisors [19]. In addition, the relationship between supervisors and GP registrars can have a role in the process of practicing EBM. For example, in the current study, some registrars stated that supervisors wanted them to follow their advice without critically evaluating it. This could be a challenging situation for GP registrars because of the differential power and experience between registrars and supervisors. Similar to the current finding a study on barriers for practicing EBM among GP registrars also identified that registrars might lose their motivation to seek an answer for their clinical questions if the supervisor does not provide them sufficient autonomy [20].

Thus, further research might examine the effect of courses that incorporate discussion of what might GP registrars face in practice and how to deal with it. Research on interventions for GP supervisors to facilitate and encourage the practice of EBM by their registrars could also be beneficial. Given that discussion sessions between registrars and supervisors are an opportunity for registrars to learn, collaborative learning discussions can be utilised for development and translation of EBM skills into practice while creating a safe, equal and collaborative environment between registrars and supervisor [42]. A previous study reported that this approach was useful for promoting EBM [42].

The effectiveness of future EBM training courses might be improved by providing strategies for responding to patient preferences that are at odds with research evidence. For example, a non-randomised controlled trial showed that GP registrars, who took part in online training on improving communication with patients, prescribed significantly fewer antibiotics compared to the control group [43].

### Relevance of theory to the study findings

As the objective of the course was to change behaviours of registrars, we considered our results in relation to two frameworks developed for behaviour change. We first compared our findings with theoretical domains proposed to identify the process specifically involved in changing the behaviour of the health professionals for practicing EBM [44]. This framework identified specific domains that were directly relevant to the implementation of EBM training. Then, we applied an overarching framework -the behaviour change wheel (BCW)- which had been developed for improving the design and implementation of interventions for behaviour change in general [45].

Michie et al. (2005) reviewed the evidence on motivation theories (e.g. Social Cognitive Theory), action theories (e.g. Learning Theory and Organisation Theories (e.g. Goal Theory; Michie et al., 2005) for explaining the process of behaviour change. Twelve domains were identified, nine of which were relevant to our findings. Table 3 illustrates our study findings across these nine domains.

**Table 3:** Theoretical domains underpinning the study findings

Domains*	Study findings	
	Perceived impact	Barriers and enablers
Knowledge	Increase in knowledge of Finding/interpreting/critiquing research information	Technical issues
Skills/ability	Ability to Find/ interpret/critique/ use research information	
Social/professional role and identity (Self-standards)		GPs self-standards ensured to choose the high-quality and EBM guideline
Beliefs about capabilities (self-confidence)	Increased confidence	
Beliefs about consequences (Anticipated outcomes/ attitude)	Awareness of the importance of incorporating research into practice	Perception about EBM, considering supervisor recommended practice to be acceptable without questioning
Memory, attention and decision processes		Difficulty remembering to use research evidence
Environmental context and resources	Increased access to databases	Time, access to databases, system
Nature of the behaviours (Routine)		Past (existing) behaviour
Social influences (Norms)		Supervisors and accepted norms

*Michie et al. 2005 [44]

In addition, relating the behaviour change wheel (BCW) framework [45] with our findings suggests that the course was able to address two out of three essential conditions of ‘capability’, ‘opportunity’, and ‘motivation’ required for behaviour change. By providing two intervention functions (education and training), the course increased the GP registrars’ knowledge, understanding and skills of EBM. Consequently, it increased the registrar’s ‘capability’ (physical and psychological) to practice EBM. It also provided an ‘opportunity’ (physical) for registrars to practice what they had learnt by being implemented when learners were exposed to clinical cases (environmental restructuring). However, the conditions of ‘motivation (both automotive and reflective)’ and the ‘psychological opportunity’ were not identified as part of the course impact. According to the BCW framework, the two intervention functions of modelling and persuasion (using communication to induce positive or negative feelings) can be used to increased automatic and reflective motivation [45]. Considering the influence of supervisors on registrars [19], they can serve as a role model for registrars to deliver modelling function. In addition, by reducing barriers and increasing means (e.g. behavioural support) the future course can increase opportunities and reflective motivation [45].

### Implications

The current study suggests an engaging online course that is relevant to GP’s daily practice can be used as appropriate training for EBM among GP registrars. Supervisors could have a considerable impact on registers’ motivation and ability to translate EBM skills into practice by encouraging registrars and actively engaging in an equal, interactive discussion around EBM. Future studies might explore the addition of simultaneous supervisor training. In addition, future EBM learning courses might consider training registrars on how to respond to patients if their preferences are at odds with research evidence. Our findings were consistent with Mitchie’s nine domains suggested for behaviour change of health professionals. It suggests that online course may be effective if improves knowledge, skills/ability, confidence and beliefs about consequences. Some factors may act as barriers or enablers such as how GPs see themselves in their professional role, memory, routine, social influences or environmental context and resources.

### Limitations

There are several limitations to this study that should be acknowledged. The study only provided data on learner perspectives after finishing the course. Objective measures of knowledge, attitudes and behaviours were not collected from a control group that received no training or training with different type of educational modality. Also, data were notcollected before the intervention to measure changes over time. Consequently, the study is vulnerable to social desirability (telling us what we want to hear) and recall biases.

The online survey achieved a response rate of only 34%, which does bring issues of responder bias. This is, however, quite a reasonable response rate for a survey of GPs without incentives [46].

The telephone interviews sample was homogeneous and lacking in a full spectrum of variation. In particular, only one student was trained overseas, and the experience of overseas-trained students could be different to those of locally trained students. Since the data collection ceased before the data saturaation, we might have missed some information. However, there was good saturation in most of the themes. Further, the study used triangulation to compare results from the survey and qualitative interviews and found them generally consistent.

## Conclusion

This study showed that an online interactive and multi-media training that aimed to enable GP registrars to use research evidence in their practice changed registrars’ knowledge, confidence, skill and behaviour of EBM. The findings of this study can assist future educational interventions to be effective. We conclude that an interactive and engaging online course with relevant practical scenarios can be effective in teaching EBM. Further, this study highlights the importance of the supervisor’s role in GP registrars’ ability in translating the EBM skills learnt in to practice. Thus, studies might want to explore how educating supervisors can facilitate their role in supporting registrars to practice EMB. Incorporating training on how registrars can engage with patients regarding evidence might also be considered in future EBM course developments. In addition, considering theoretical frameworks for developing interventions suggest that investigating the role of motivation, emotion and behavioural change techniques on practicing EBM might be beneficial.

## Supplementary Information

**Additional file 1 Table S1** Topics, lessons and learning objectives of the six modules. **Fig. S1** Examples of the course screens. **Table S2** Response to an open-ended question on what participants liked most about the course (*n* = 160). **Table S3** Responses to an open-ended question on what participants would like to see changed in the course (*n* = 98).

## Data Availability

The datasets generated and analysed during the current study are not publicly available because consent was not obtained from study participants for data to be made public but are available from the corresponding author on reasonable request subject to approval from the Human Research Ethics Committee at UNSW Sydney.

## Abbreviations

EBM: Evidence-based medicine
GP: general practitioner
PBS: the Pharmaceutical Benefits Scheme
