# Supplementary material for: Online training in evidence-based medicine and research methods for GP registrars: a mixed-methods evaluation of engagement and impact
Source: BMC Med Educ. 2021 Sep 14;21:492. doi: 10.1186/s12909-021-02916-0 (PMC8439372; doi:10.1186/s12909-021-02916-0)
Supplement: Supplementary file 1 — Additional file 1 Table S1 Topics, lessons and learning objectives of the six modules. Fig. S1 Examples of the course screens. Table S2 Response to an open-ended question on what participants liked most about the course (n = 160). Table S3 Responses to an open-ended question on what participants would like to see changed in the course (n = 98). [file 12909_2021_2916_MOESM1_ESM.docx]

**Online training in evidence-based medicine and research methods for GP Registrars: a mixed-methods evaluation of engagement and impact**

# Additional file 1

Table S1 Topics, lessons and learning objectives of the six modules

| **Module topics and lessons** |
| --- |
| **Topic 1: Levels of engagement in critical thinking and research and ethical principles in human research** |
| 1.1 How do you answer clinical questions? |
| 1.2 Problems with eminence-based medicine |
| 1.3 Evidence-based medicine |
| 1.4 Research quality |
| 1.5 GP involvement in research |
|  |
| **Topic 2 Critical appraisal skills** |
| 2.1 Introduction and the PICO approach |
| 2.2 Searching for the evidence |
| 2.3 Appraising a study |
| 2.4 Pragmatic searches |
|  |
| **Topic 3 Basics of quantitative research methods** |
| 3.1 Quantitative study designs |
| 3.2 Sampling strategies |
| 3.3 Data analysis and interpretation |
|  |
| **Topic 4 Principles of qualitative research** |
| 4.1 Introduction to qualitative methods |
| 4.2 Methods of qualitative research |
| 4.3 Critical appraisal |
|  |
| **Topic 5 Exploring the evidence** |
| 5.1 Systematic reviews in clinical practice |
| 5.2 The systematic review process & meta-analysis |
| 5.3 Using systematic reviews and meta-analyses in your clinical practice |
|  |
| **Topic 6 Understanding clinical epidemiology** |
| 6.1 Defining clinical epidemiology |
| 6.2 Principles: Causation |
| 6.3 Principles: Diagnostic testing |
| 6.4 Applications of clinical epidemiology |


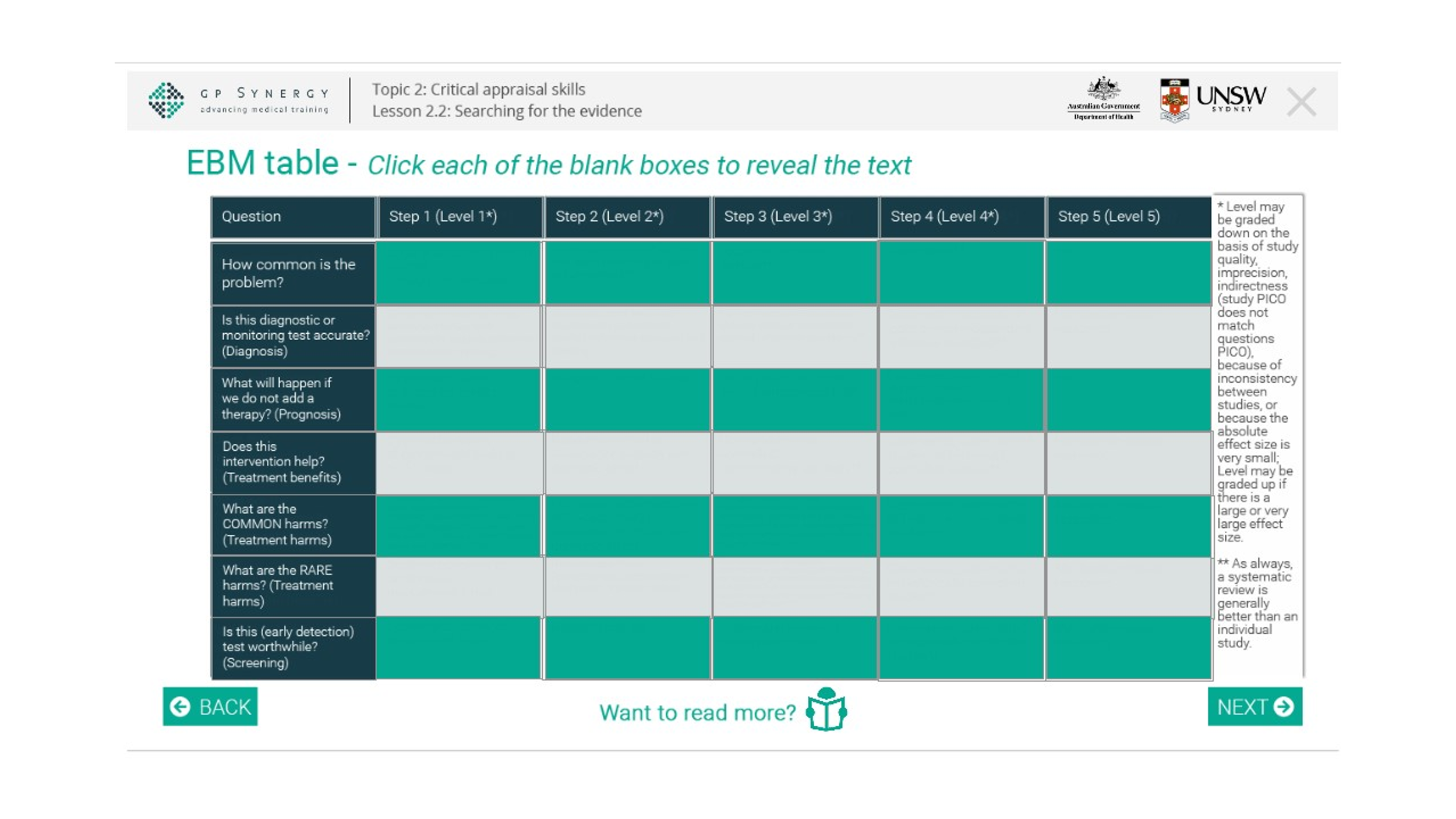

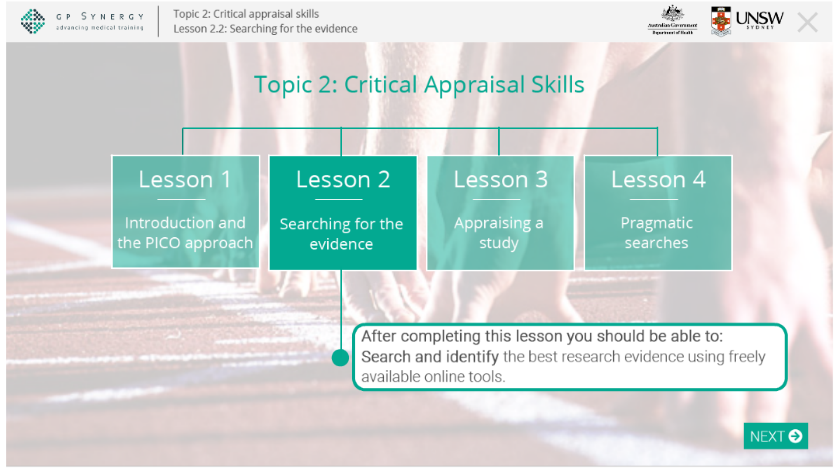

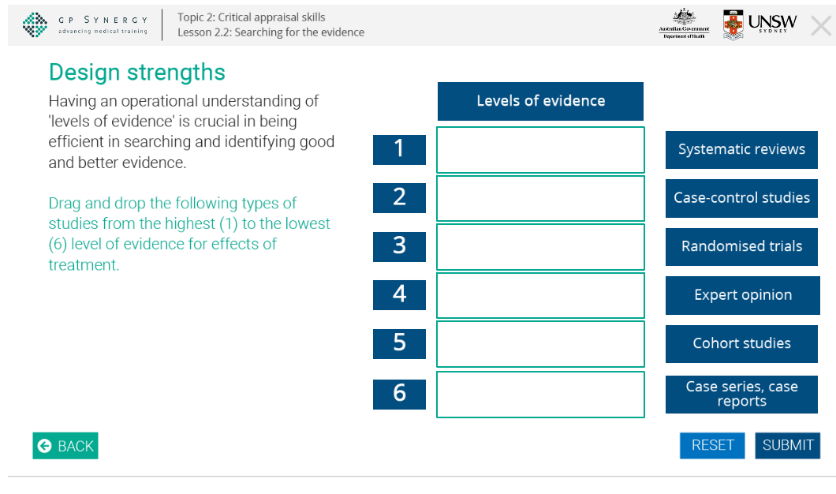

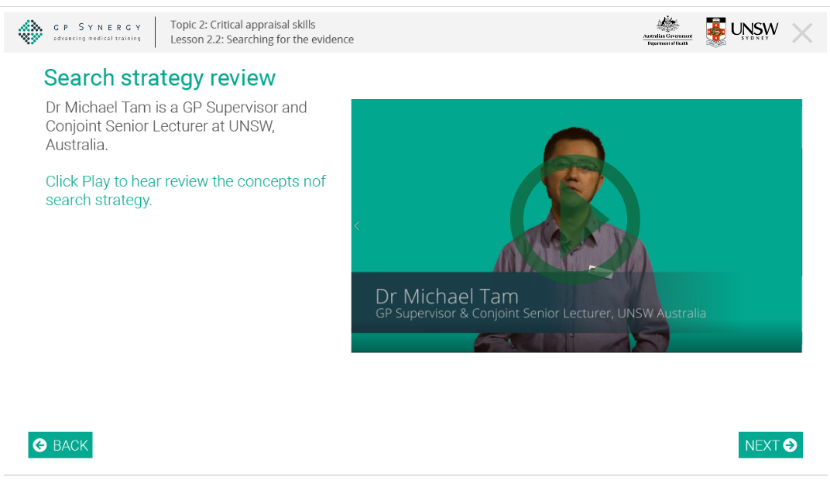


Figure S1. Examples of the course screens

Table S2 Response to an open-ended question on what participants liked most about the course (n=160)

| Categories | Frequency |
| --- | --- |
| **Topic** |  |
| Clinical epidemiology | 17 |
| Resources such as database resources | 10 |
| Search skills | 8 |
| Qualitative research | 3 |
| Quantitative research | 3 |
| Evidence-based medicine | 3 |
| Systematic review | 3 |
| Critical appraisal | 1 |
| Content (overall - topics not specified) | 7 |
| **Content** |  |
| Easy to understand | 23 |
| Examples/relevant examples | 23 |
| The videos e.g. Ted talks, YouTube | 23 |
| Relevant to practice | 19 |
| Brief/ short lessons | 12 |
| Comprehensive | 10 |
| Good revision | 5 |
| **Delivery** |  |
| Self-paced/flexible with time | 42 |
| Interactivity including quiz and feedback | 23 |
| Structure | 12 |
| User friendly | 11 |
| Being online/accessible | 9 |
| Varied modes of learning (video, text, quizzes) | 3 |
| Other | 6 |

Table S3 Responses to an open-ended question on what participants would like to see changed in the course (n=98)

| **Categories** | **Frequency** |
| --- | --- |
| **Content** |  |
| Increase relevance to practice | 9 |
| Simplify the qualitative module | 7 |
| More detail on basic statistics | 3 |
| **Interface** |  |
| Less technical issues | 56 |
| More user-friendly (e.g. ability to see the progress throughout out the course) | 28 |
| **Quality and layout** |  |
| Briefer | 12 |
| More quizzes (particularly at the end of each module) | 8 |
| Fact sheets and takeaway messages/summary | 7 |
| More relevant examples | 5 |
| Less repetition of what is already known | 5 |
| More engaging (i.e. interesting) | 4 |
| Less negative questions | 4 |
| Simpler | 3 |
| Clearer wording in questions and answers | 3 |
| Other | 3 |
